# The Coping Strategies and Cumulative Changes in Intensive Care Unit Nurses after Experiencing Professional Grief: A Hermeneutic Phenomenological Study

**DOI:** 10.1155/2024/3682609

**Published:** 2024-02-19

**Authors:** Zhengmin Zhang, Manyi Gao, Zejian Fang, Xin Chen, Qiaoqiao Shen, Yulin Gao

**Affiliations:** ^1^School of Nursing, Southern Medical University, Guangzhou, Guangdong, China; ^2^Guangdong Mechanical & Electronical Polytechnic, Gaungzhou, Gaungdong, China; ^3^School of Chinese Medicine, Southern Medical University, Guangzhou, Guangdong, China

## Abstract

**Background:**

Compared to nurses in other hospital departments, intensive care unit (ICU) nurses have more frequent exposure to patient deaths, potentially rendering them more susceptible to experiencing professional grief following patient fatalities.

**Objective:**

To explore the coping experiences of ICU nurses following their encounter with professional grief.

**Methods:**

This study utilized a qualitative research design based on Heideggerian phenomenology. A purposive sample of 18 ICU nurses was selected from six tertiary hospitals in Guangdong Province, China. Individual semistructured interviews were audio-recorded and transcribed verbatim. The transcribed texts were analyzed using interpretative phenomenological analysis.

**Results:**

Two main themes emerged: (1) short-term dual coping with professional grief and (2) long-term cumulative changes from professional grief. These themes reflect the dynamic coping processes experienced by ICU nurses amidst frequent encounters with loss and grief during their clinical work.

**Conclusions:**

ICU nurses employ both adaptive and maladaptive coping strategies to address professional grief, significantly impacting their personal and professional well-being. It is advisable to offer targeted education and organizational support systems for ICU nurses to promote positive cumulative growth when they repeatedly encounter patient deaths.

## 1. Introduction

Professional grief, within the healthcare context, refers to the emotional distress experienced by healthcare practitioners following patient deaths during clinical practice [1]. This distress often presents with symptoms such as sadness, self-blame, insomnia, decreased appetite, and intrusive thoughts about death [1]. Due to the unique nature of the relationship between healthcare professionals and patients, the professional grief they undergo, distinct from familial grief, is often characterized as disenfranchised grief—not widely recognized or accepted by the general public [2]. Data from the Societies of Intensive and Critical Care reveal that the global average mortality rate for intensive care unit (ICU) inpatients falls within the range of 10% to 29% [3]. Compared to their counterparts in other hospital departments, ICU nurses are more frequently exposed to patient deaths, making them more susceptible to experiencing professional grief [4].

Existing research emphasizes the inevitability and prevalence of professional grief among healthcare professionals [1, 4]. For example, Papadatou et al. [5] conducted semistructured interviews with 63 oncology and ICU nurses caring for terminally ill children in Greece and Hong Kong, revealing that 93% of nurses had experienced grief following patient deaths. Faced with professional grief, many healthcare professionals resort to maladaptive coping strategies, such as avoiding direct exposure to death and death-related environments [6, 7], evading communication with patients' families [6, 7], and turning to substance abuse to manage psychological stress [8]. While some studies investigate adaptive coping mechanisms employed by healthcare professionals in professional grief, such as openly expressing their emotions [9, 10], engaging in recreational activities [7, 10], or seeking support from family [7, 9, 10], these studies lack an in-depth exploration of the psychological coping mechanisms used by healthcare professionals in professional grief and the potential long-term impacts on their personal and professional lives.

To date, the majority of research on professional grief among nurses has been concentrated in Europe and North America, with relatively limited participation from non-Western regions [1]. In China, the nursing profession faces challenges such as low societal recognition, demanding workloads, and strained nurse-patient interactions [11]. Consequently, the professional grief experienced by nurses is often marginalized or inadequately addressed [10]. This study employed a phenomenological research approach with the aim of comprehensively analyzing the coping experiences of Chinese ICU nurses in the face of professional grief. The goal is to enhance the conceptual model of professional grief among healthcare professionals in a cross-cultural context and facilitate managers in devising intervention strategies to address professional grief.

## 2. Methods

### 2.1. Study Design

This phenomenological study was conducted within the hermeneutic paradigm proposed by the German philosopher Martin Heidegger (1889–1976) [12]. Heidegger's perspectives primarily focus on elucidating the essence of existence, emphasizing an understanding of the modes of existence and the intrinsic meaning of existence itself, rather than solely concentrating on the surface phenomena of objects [13]. To facilitate operational procedures, we employed Jonathan A. Smith's developed method known as the Interpretative Phenomenological Analysis (IPA) framework [14]. The reporting of this study adhered to the Consolidated Criteria for Reporting Qualitative Research (COREQ) checklist [15].

### 2.2. Study Participants

From October 2020 to May 2021, ICU nurses were recruited through purposive sampling from six tertiary hospitals in Guangdong Province, China. Inclusion criteria comprised possessing a valid nursing license, a minimum of 6 months of ICU experience, and previous exposure to patient deaths. Exclusion criteria included refusal to participate in the study, being on vacation, and the presence of severe physical or mental illnesses. IPA research involves a meticulous analysis of cases, typically conducted within a small sample size of around 10 cases [14]. Smith et al. also suggested that determining the sample size should be pragmatic, considering the depth and breadth of the study, rather than adhering to fixed standards [16]. Following the fundamental principle of “data saturation” [17], researchers conducted in-depth interviews with 12 ICU nurses from a single hospital. Subsequently, interviews were extended to include 6 ICU nurses from five additional hospitals, concluding recruitment upon confirmation that no new themes or codes emerged.

### 2.3. Data Collection

The first and second authors conducted individual face-to-face interviews with participants in a quiet lounge after their work hours. The interview guide, developed through a literature review, expert consultation, and pilot interviews with four ICU nurses, included the following questions: (1) Could you share your most memorable experience involving a deceased patient during your ICU work? (2) What internal feelings arise when confronted with patient deaths? (3) Have you employed strategies to alleviate these feelings, and how effective were they? (4) When saddened by a patient's passing, what support mechanisms are available to you? (5) How have your reactions and coping strategies evolved since your initial encounter with patient deaths? The interviews varied in duration from 42 to 96 minutes, with an average interview time of 58 minutes. All interviews were recorded and transcribed verbatim. Both interviewers meticulously reviewed the transcripts for accuracy, providing interviewees with the opportunity to verify and validate their statements.

### 2.4. Data Analysis

The analytical process was subjected to IPA, wherein a meticulous idiographic analysis was prioritized on a case-by-case basis, followed by the exploration of patterns across cases [16]. Following the six-stage process outlined in the IPA research guidelines [14], each complete transcript was thoroughly and repeatedly read line-by-line, annotated to develop emergent themes and explore connections between emerging themes. This process was iteratively applied to review all remaining cases, identifying patterns across cases and determining themes and subthemes reflecting the experiences of all participants. The first author led the analysis, with each step reviewed and verified by the corresponding authors. The final analysis underwent scrutiny by all other team members, ensuring consensus on the overall interpretation of interviews and contributing to the ultimate findings. NVivo version 12.0 was utilized for data management and analysis assistance.

### 2.5. Trustworthiness/Credibility

The first author, who has previously interned in the ICU, established positive relationships with participants, facilitating the sharing of sensitive experiences related to professional grief. Before formal interviews, researchers extensively practiced using the interview guide and conducted pilot interviews to refine their interview skills. Both the first author and corresponding authors received standardized training, possessed rich qualitative research experience, and employed a systematic approach to iteratively analyze transcripts. Throughout the analysis process, researchers engaged in continuous self-reflection, questioning data, and existing knowledge to ensure the validity of interpretations without undue stretching or overinterpretation. In addition, they actively sought review and validation from team members and participants to ensure the accuracy and reliability of the study results.

### 2.6. Ethical Considerations

The study received ethical approval from the Southern Medical University Ethics Committee (No. 2020−16). Participants were informed about the study's purpose and gave written informed consent. Participant identities are anonymized using numerical codes. Audio recordings and transcribed data are securely stored in password-protected files, accessible only to research team members.

## 3. Results

### 3.1. Participant Characteristics

A total of 18 ICU nurses, comprising nine males and nine females, participated in this study. The participants' ages ranged from 23.0 to 37.0 years (mean age of 30.0 years), and their average tenure in ICU nursing was 7.3 years. Among the participants, 4 were supervisor nurses, and 1 held a master's degree. None of the participants reported having any religious beliefs, and none had received professional training related to grief adjustment. The characteristics of the participants are presented in Table 1.

### 3.2. Emerging Themes and Subthemes

The coping experiences of ICU nurses with professional grief were identified as two themes, comprising six subthemes and 42 codes. The results are presented in Table 2.

#### 3.2.1. Short-Term Dual Coping with Professional Grief

*(1) Avoidance of Death and Confrontation with Death*. For the majority of ICU nurses, the experience of encountering patient deaths was a significantly stressful event. Consequently, they often chose to avoid direct contact with dying or deceased patients, refrained from interacting with patient families during mourning scenes and sidestepped discussions regarding patient death.

*N4: “My main focus was on the medical condition, and I preferred not to delve into discussions about information connected to patient death.”*

*N8: “I didn't want patients to pass away while I was on duty. If I could hand over the shift to someone else, it was a relief.”*

*N15: “I chose to avoid, trying not to hear the cries of the family members.”*

In contrast, some nurses attempted to derive meaning from patient deaths by rationalizing them as liberation, finding solace in destiny-based explanations for death, or believing themselves to have fulfilled their caregiving responsibilities. Moreover, a few nurses chose to bid farewell to deceased patients, accompanying them through their final moments as a means of completing the mourning process.

*N1: “He's finally at peace, no more suffering.”*

*N7: “I just stood there silently, watching her, accompanying her through the final moments of her life...”*

*N13: “Everyone has their own fate; you have to believe in it.”*

*N18: “I feel like I have a clear conscience; I've provided the best care to the patient.”*

*(2) Psychological Detachment and Psychological Adjustment*. Three nurses reported proactively disengaging from their duties and taking a break in the relaxation area after completing postmortem care. Simultaneously, the majority of ICU nurses actively disengaged from work after their shifts, deliberately avoiding contemplation of work-related matters to prevent further interference with feelings of grief.

*N3: “when I'm off duty, I don't want to bring these emotions home.”*

*N16: “As I left the ward environment and entered the living area, I gradually underwent a process of detachment.”*

Nevertheless, participants also recognized that, at times, they faced challenges in completely disengaging from the negative emotions associated with patient deaths. In these instances, they made efforts to mitigate their grief through distraction techniques, including engaging in conversations, participating in leisure activities, or indulging in retail therapy.

*N12: “I tried chatting with colleagues or doing something else, just to quickly divert my attention.”*

*N13: “I went for walks, watched movies, and did some shopping, all just to forget about these things.”*

*(3) Social Withdrawal and Support Seeking*. After experiencing the death of a patient, some ICU nurses exhibited a strong desire for solitude. They temporarily withdrew from social and recreational activities, declined unnecessary interactions with others, and lost interest in the external world.

*N2: “The entire world seemed gray; I became quieter. Even when I walked on the street after work, I didn't want to look around.”*

*N6: “I didn't feel like talking; I just wanted to find a quiet place to be alone, and it was better if others didn't bother me.”*

ICU nurses had often attempted to seek support from colleagues and family members to express or release their own grief. However, this support sometimes remained on the surface, and inappropriate consolation or less-than-ideal reactions left ICU nurses feeling isolated.

*N6: “I used to talk to my mom when I first started working, but later she said, 'Why is your department so scary?' so I stopped talking to her about it.”*

*N11: “I talked to the head nurse about my fears, and she said, 'You'll get used to it after seeing it more.”*

#### 3.2.2. Long-Term Cumulative Changes from Professional Grief

*(1) Cherishing Life and Existential Meaninglessness*. ICU nurses transitioned from initial anxiety about death to valuing health, embracing the present moment, and cherishing relationships with loved ones. This transformation reflects an awakening derived from their experiences of grief. They also contemplated their own death, desiring to make rational end-of-life arrangements for the future, leading towards a death with a high quality of life.

*N1: “Having witnessed patient deaths, I now understand the importance of being there for family.”*

*N9: “Taking care of my health, living each day to the fullest; who knows what the future holds? If I have a choice, I hope not to experience too much pain at the end of life.”*

Some nurses had witnessed numerous patients teetering on the edge of life and death, eventually succumbing to their fate, leading to nurses feeling a profound sense of helplessness. Faced with the fragility and unpredictability of life, these nurses engaged in deep reflections on the meaning and value of existence. This contemplation led these nurses to adopt the conviction that life is transient, potentially causing them to lose sight of life goals and perhaps slipping into a sense of existential nihilism.

*N8: “Life is so difficult, and I don't even know what people are living for.”*

*N10: “I feel like there are not many joyful things in life, and I don't want to strive for a lot of things; it doesn't seem meaningful.”*

*(2) Emotional Resilience and Emotional Exhaustion*. Most ICU nurses indicated that, after repeatedly experiencing patient deaths in their work, their emotions were not as profoundly affected as when they first started. They were able to quickly adjust their emotions within a short period.

*N3: “After putting in years on the job, I've gotten pretty good at handling my emotions on my own. It's been working out quite well.”*

*N18: “Back in the day, it used to take me over two weeks to shake off a bad mood, but these days, I can bounce back right after wrapping up my shift.”*

Moreover, several interviewees mentioned encountering emotional fatigue in the face of patient deaths. They exhibited a reduced sense of empathy towards the families of patients, experienced a decline in the intensity of professional grief, or even reported a complete absence of such feelings.

*N5: “The deaths of patients don't affect me as much anymore; I recognize that they're reaching the end, and I don't experience strong emotions about it.”*

*N9: “Having witnessed numerous resuscitations and gone through so many, I've developed emotional numbness.”*

*(3) Professional Development and Occupational Burnout*. Most ICU nurses drew lessons from cases of patient deaths, focusing on enhancing their professional skills to effectively handle similar medical situations. They also reflected on their inadequate abilities in end-of-life care and grief counseling but strove to maintain the dignity of patients and fulfill the requests of grieving families to the best of their ability.

*N8: “In most cases, we find ourselves at a loss for words when facing family members... striving to fulfill their wishes to the best of our ability.”*

*N17: “(Reflecting on a patient's death) The challenges in these situations force me to grow up fast; I become more careful and detailed in observing the medical condition.”*

In the busy environment of the ICU, nurses often found themselves too occupied to attend to their own grief. As a result, they intentionally maintained emotional distance from patients, mechanically adhering to established caregiving procedures. In addition, nurses expressed an inclination to transition to departments with reduced exposure to patient deaths or to consider leaving the nursing profession altogether.

*N1: “I feel like I'm being a robot, going through the same process day after day, taking care of one patient, and then the next, in a repetitive cycle.”*

*N10: “After working in the ICU for a long time, I want to change the work environment, dealing with patients who can move and speak.”*

## 4. Discussion

### 4.1. Main Findings

To the best of our knowledge, this is the first qualitative research conducted in mainland China that specifically investigates the professional grief experienced by ICU nurses. Our research synthesizes the dual coping strategies employed by ICU nurses within the Chinese cultural context, focusing on dealing with death, psychological adjustment, and external support. In addition, our findings highlight the cumulative impact of professional grief on both the personal and professional lives of ICU nurses, addressing the scarcity of evidence regarding ICU nurse professional grief in non-Western regions [1].

### 4.2. Comparing Study Findings with Relevant Professional Grief Models

In this study, ICU nurses commonly experienced grief due to patient deaths and employed various adaptive coping mechanisms, such as rationalizing death, bidding farewell proactively, and psychological detachment. Some nurses, however, tended to adopt maladaptive coping strategies, including death avoidance and social withdrawal, in response to professional grief, aligning with the Model of Health Professionals' Grieving Process proposed by Papadatou [18]. Nevertheless, in contrast to this model, we argue that the construction of meaning around patient death and behaviors like self-adjustment and seeking support are coping strategies for professional grief, rather than outcomes of the grieving process or consciously completed tasks. Furthermore, the cumulative effects of repeated encounters with professional grief, whether positive or negative, align with the Professional Grief Integration Model by Chen et al. [1], where the cumulative experiences of multiple patient deaths eventually have long-term personal and professional consequences for healthcare providers. In our study, while discerning shifts in the perspectives of ICU nurses on life and death, there is minimal discourse regarding transformations linked to religious beliefs. In contrast to Western nations, the religious ambiance in Chinese society is relatively subdued [10]. Embedded within traditional Chinese culture is the notion that “life and death are predestined, and wealth and poverty are heaven's arrangement,” significantly influencing individuals' attitudes towards life and death [10]. This inclination tends to foster a preference for embracing reality over seeking solace through religious faith.

### 4.3. ICU Nurses Lacking Support to Cope with Professional Grief

After experiencing patient deaths, ICU nurses frequently found themselves confronted with a shortage of adequate professional resources to cope with their grief—a situation pervasive in many healthcare systems [6, 19]. In response, they often resorted to employing self-distraction strategies, suppressing their grief and emotions to fulfill their professional duties [20]. While this self-protective mechanism may offer some short-term relief, the accumulation of unaddressed grief over time poses the risk of leading to professional grief overload among ICU nurses [21]. Despite their attempts to confide in colleagues or family members for support, the cultural taboo surrounding death often stigmatizes discussions about mortality and grief, deeming them as inauspicious or inappropriate topics [10]. This, in turn, leaves nurses feeling isolated and helpless, deprived of the emotional support they urgently require [10]. In light of these challenges, it becomes imperative for healthcare institutions to prioritize the monitoring of the emotional well-being and psychological state of ICU nurses and to provide resources and channels that facilitate professional grief relief for them [21].

### 4.4. ICU Nurses Undergoing Negative Cumulative Changes from Professional Grief

This study revealed that ICU nurses, when exposed to prolonged instances of patient deaths, tended to experience emotional exhaustion and a decline in empathy towards both patients and their families. Some ICU nurses adopted a strategy of limiting emotional involvement, delivering care in a more mechanical manner to alleviate professional grief. While this approach can mitigate professional grief to some extent, it may lead to an insufficient provision of humanistic care for patients and their families, a decrease in nursing quality, and diminished job satisfaction among ICU nurses, ultimately resulting in a loss of nursing talent [22]. Our research found that these adverse cumulative changes were notably influenced by maladaptive coping strategies employed by ICU nurses in the face of patient deaths. Conversely, adaptive coping strategies resulted in a positive perspective on life and death, improved interpersonal relationships, and significantly enhanced self-emotional regulation abilities. Therefore, it is crucial to acknowledge the emotional shifts and psychological states experienced by ICU nurses, especially those with limited experience [23], and guide them in redefining the meaning of life and death through their losses and fostering personal reflection and growth.

### 4.5. Study Limitations

This study has several limitations. First, being a phenomenological study, our objective is exploratory rather than confirmatory, delving into the in-depth examination of grief coping experiences among ICU nurses—experiences that often go unnoticed. To ascertain variations in their professional grief coping and its evolutionary processes, further investigation with a larger sample of ICU nurses is imperative. Second, the researchers, serving as the investigative tool, may introduce interviewer bias. To ensure credibility, we engaged in self-reflection, avoiding the impact of a priori assumptions, and employed the triangulation method for data collection and analysis [24]. Third, although interviews were conducted in 2021, data analysis was not completed until 2023, potentially resulting in a lag in data information. Fourth, our interviews took place during the COVID-19 pandemic. During this unique period, strict infection control measures were in place, and healthcare professionals experienced increased workloads. These factors may have affected participants' availability and psychological resilience, potentially making them feel more stressed or fatigued during the study. Finally, our study was conducted exclusively in Guangdong Province, China. While our findings align closely with research on healthcare professionals' professional grief in other regions, caution should be exercised when generalizing the results.

## 5. Conclusion

In summary, ICU nurses employ adaptive or maladaptive strategies to navigate professional grief, resulting in subsequent positive or negative cumulative changes in both their personal and professional lives. It is imperative to guide ICU nurses in recognizing and expressing their professional grief, provide formal organizational support, and offer professional psychological counseling to promote their adaptive coping and personal growth. Further research with larger sample sizes and across multiple regions is recommended to develop and evaluate interventions facilitating ICU nurses' coping with grief when confronted with patient death.

## Figures and Tables

**Table 1:** Characteristics of study participants (*n* = 18).

Number	Gender	Age, years	Employment duration in the ICU, years	Positional title	Educational background	Marital status	Psychological acceptance of patient deaths
N1	Female	31	5	Senior nurse	College degree	Unmarried	Moderate
N2	Female	24	2	Registered nurse	College degree	Unmarried	Moderate
N3	Male	30	8	Senior nurse	Bachelor's degree	Married	Unacceptable
N4	Male	32	8	Senior nurse	College degree	Married	Somewhat acceptable
N5	Female	25	4	Registered nurse	College degree	Unmarried	Somewhat acceptable
N6	Female	36	16	Senior nurse	Bachelor's degree	Married	Moderate
N7	Male	37	14	Nurse supervisor	College degree	Unmarried	Moderate
N8	Female	28	8	Supervisor nurse	Bachelor's degree	Married	Moderate
N9	Female	27	1	Senior nurse	College degree	Married	Moderate
N10	Female	37	13	Senior nurse	College degree	Married	Somewhat acceptable
N11	Male	32	11	Senior nurse	Bachelor's degree	Married	Somewhat acceptable
N12	Female	35	14	Supervisor nurse	Bachelor's degree	Married	Moderate
N13	Male	23	3	Registered nurse	College degree	Unmarried	Somewhat acceptable
N14	Male	25	3	Registered nurse	College degree	Unmarried	Moderate
N15	Male	28	4	Registered nurse	Bachelor's degree	Unmarried	Moderate
N16	Female	34	10	Supervisor nurse	Master's degree	Married	Moderate
N17	Male	27	2	Registered nurse	Bachelor's degree	Unmarried	Somewhat acceptable
N18	Male	29	6	Senior nurse	Bachelor*'*s degree	Unmarried	Moderate

**Table 2:** Themes, subthemes, and codes.

Themes	Subthemes	Codes
Short-term dual coping with professional grief	Avoidance of death and confrontation with death	(i) Avoidance of deceased patients/avoidance of dying patients/avoidance of patient families/avoidance of mourning scenes/avoidance of discussions on death
		(ii) Patient's relief from suffering/family's relief from distress
		(iii) Fate's interpretation
		(iv) Clear conscience/voluntary farewell/voluntary mourning
	Psychological detachment and psychological adjustment	(i) Maintaining distance from work/forgetting work-related matters/taking a short break
		(ii) Relaxing oneself/shifting attention
	Social withdrawal and support seeking	(i) Self-isolation/diminished interest
		(ii) Family support/friend support/colleague support/leadership support

Long-term cumulative changes from professional grief	Cherishing life and existential meaninglessness	(i) Valuing health/valuing quality of life/changing priorities/enjoying the present/spending time with family
		(ii) Worthlessness of life/meaninglessness of life/lack of life goals
	Emotional resilience and emotional exhaustion	(i) Familiar situation/inner calmness/rapid adjustment
		(ii) Emotional numbness/empathic fatigue/emotional absence
	Professional development and occupational burnout	(i) Medical knowledge and skills/end-of-life care/grief counseling
		(ii) Depersonalization/reduced accomplishment/intentions to leave

## Data Availability

The datasets analyzed during the current study are not publicly available due privacy issues of participants but are available from the corresponding author on reasonable request.
